# Regional climate variation structures the phyllosphere microbiome of flue-cured tobacco

**DOI:** 10.3389/fpls.2025.1733198

**Published:** 2026-01-15

**Authors:** Cheng Zhang, Lei Yang, Xiaohua Zhang, Yuhang Zhao, Jiati Tang, Zhijun Cheng, Yi Cao, Shengjiang Wu, Guanhui Li, Long Yang, Kesu Wei

**Affiliations:** 1 College of Plant Protection, Agricultural Big-Data Research Center and Key Laboratory of Agricultural Film Application of Ministry of Agriculture and Rural Affairs, Shandong Agricultural University, Tai’an, China; 2 Guizhou Academy of Tobacco Science, Guizhou Provincial Academician Workstation of Microbiology and Health, Guiyang, China; 3 China Tobacco Hunan Industrial Co., Ltd, Changsha, China

**Keywords:** climate adaptability, co-occurrence network, functional prediction, phyllosphere microbiota, tobacco

## Abstract

**Introduction:**

Climate change poses major challenges to agriculture, with phyllosphere microbiota playing a key but poorly understood role in plant adaptation.

**Methods:**

We examined structural and functional responses of the phyllosphere microbiome in flue-cured tobacco (*Nicotiana tabacum* L.) across climatic gradients, using multi-regional sampling, high-throughput sequencing (16S rRNA/ITS), and functional prediction (PICRUSt2).

**Results:**

Leaf starch, total sugar, and reducing sugar contents varied significantly (26.87–32.25%, 14.24–16.74%, and 9.96–11.26%, respectively). Bacterial communities were primarily shaped by precipitation (41.7% variance explained), whereas fungal communities were mainly driven by temperature (27.3%). Microbial networks showed climate-adaptive patterns: complex, cooperative networks (85.99% positive edges) in high-precipitation areas versus simplified, drought-tolerant networks (Nodes: 93, Edges: 1124) dominated by *Sphingomonas* (86.50%) and *Methylobacterium* (10.24%) in arid regions. Metabolic potential shifted along the gradient: Communities in low-precipitation areas were enriched with genes potentially encoding starch-degrading enzymes (e.g., α-amylase), while those in high-precipitation areas showed enhanced potential for sucrose synthesis (e.g., via sucrose synthase).

**Discussion:**

This study reveals the adaptive strategies of phyllosphere microbial communities in response to climate variations. Under low-rainfall conditions, community metabolism shifts toward starch degradation, which may aid host osmoregulation. In contrast, under humid conditions, dominant taxa such as Sphingomonas and Methylobacterium collaboratively enhance sucrose synthesis. This metabolic reprogramming aligns with structural changes in microbial networks: transitioning from complex, cooperative networks in humid regions to simplified, stress-tolerant networks in low-rainfall areas. Key metabolic functions are primarily contributed by low-abundance taxa, suggesting the vital role of the rare biosphere in maintaining functional redundancy. Based on these findings, we propose that microbial communities may enhance adaptability by retaining core metabolic functions, such as starch degradation, within rare taxa when facing drought or temperature fluctuations. This study provides a theoretical framework for improving crop climate resilience through microbiome management.

## 1 Introduction

The microbial communities on plant leaves, including bacteria, fungi, archaea and viruses, form an important interface between the plant host and its environment (Vorholt, 2012; Sohrabi et al., 2023). They are not only renowned for promoting plant growth, but also for enhancing nutrient absorption and the production of plant hormones (Zhang et al., 2021), but also receive much attention for enhancing the host’s resistance to abiotic and biotic stresses (Madhaiyan et al., 2015; Zhang et al., 2021; Misu et al., 2025). The composition and structure of these communities are highly dynamic and are greatly influenced by climatic conditions, which is one of the most crucial environmental drivers among them (Vacher et al., 2016; Stone and Jackson, 2019; Wang et al., 2023). A large amount of evidence indicates how individual climate factors shape the microbial communities in the leaf litter. Precipitation directly reshapes the community structure through physical erosion (Stone and Jackson, 2019), usually reducing the diversity of bacteria in mangroves (Wang et al., 2023), and altering the abundance of groups such as the *Methylobacterium* genus and *Pseudomonas* genus in crops like cucumber and tomato (Allard et al., 2020). Temperature indirectly affects the microbial community through regulating the host’s metabolic activities (Aydogan et al., 2018; Wang et al., 2023). Long-term warming leads to an increase in the abundance of pathogenic fungi in the leaves of poplar trees (Bálint et al., 2015), while the phyllosphere bacterial community in rice shows higher network complexity under high temperatures (Yuan et al., 2021). Light length affects the secretion of photosynthetic products from leaves, thereby changing the composition of carbon sources available to microorganisms (Vorholt, 2012). *Methylobacterium* in citrus leaves can assimilate methanol as a carbon source for other members (Yuan et al., 2025). Light induces the accumulation of secondary metabolites such as jasmonic acid in plants, indirectly selecting for tolerant microorganisms such as *Brevicoccum* sp (Huang et al., 2024). These factors do not act independently; instead, their interactions drive complex ecological patterns, such as seasonal shifts in functional groups, where summer conditions favor the growth of antagonistic bacteria like *Pseudomonas* and *Bacillus*, but also increase the risk of fungal diseases (Malik and Bouskill, 2022).

In the context of climate change, it is particularly important to understand the response mechanisms of phyllosphere microorganisms to environmental factors, as well as the patterns of their influence on the growth and development of host plants and the formation of plant quality (Zhu et al., 2022; Yuan et al., 2025; Zeng et al., 2025). The synergistic or antagonistic effects of climate factors can influence the functions of phyllosphere microorganisms by altering the physiological state of the host. For instance, under combined stress of drought and high temperature, the carbon-nitrogen ratio of plant leaves increases, leading to a decrease in the diversity of phyllosphere bacteria, but the *Firmicutes* which is drought-tolerant, enhances colonization by secreting extracellular polysaccharides (Zhu et al., 2022). Conversely, increased precipitation may promote the spread of pathogenic fungi, but simultaneously weaken the competitive ability of bacterial communities (Malik and Bouskill, 2022). Moreover, climate change specifically enriches functional microorganisms. For example, during seasonal transitions, Methylobacteria in citrus phyllosphere communities can assimilate methanol to provide a carbon source for other members (Yuan et al., 2025), while Gram-positive bacteria such as the *Actinobacteria* and *Firmicutes*, due to their thicker peptidoglycan layers in the cell walls, exhibit higher drought tolerance and alleviate the drought stress on plants (Zeng et al., 2025). These response mechanisms highlight the ecological adaptability of phyllosphere microorganisms under climate change. Therefore, in-depth studies on the relationship between climate factors and phyllosphere microorganisms are of great significance for predicting the impact of climate change on terrestrial ecosystems.

This study uses the leaf margins of flue-cured tobacco as a model system to explore these various issues. Our objectives are: (1) to determine the climatic factors that govern the assembly of dominant bacterial and fungal communities; (2) to interpret how these factors reshape the microbial co-occurrence network and community structure; (3) to elucidate the reprogramming of the metabolic potential of the resulting communities. By combining multi-site field sampling with high-throughput sequencing, network analysis, and functional prediction, this study established the mechanistic links between climatic gradients, microbial community dynamics, and host-related metabolic functions. Our research results provide a framework for developing microbiome-based strategies to enhance the climate adaptability of crops.

## 2 Materials and methods

### 2.1 Sample collection from different locations

All tobacco samples (*Nicotiana tabacum* L., variety ‘Yunyan 87’) were planted in the field in 2022, with the field transplanting date being from April 20th to 30th. On August 10th, 2022, approximately 110 days after the tobacco transplantation, fresh upper leaf samples of tobacco were collected simultaneously from six regions (**Supplementary Figure S1**), and the climate factors during the tobacco growth period in these regions were also collected (**Supplementary Table S1**). The regions were Dejiang (DJ: east longitude 108°13’, north latitude 28°25’), Fenggang (FG: east longitude 107°71’, north latitude 27°97’), Xifeng (XF: east longitude 106°62’, north latitude 26°86’), Fuquan (FQ: east longitude 107°51’, north latitude 26°70’), Huangping (HP: east longitude 107°90’, north latitude 26°90’), and Shuicheng (SC: east longitude 104°66’, north latitude 25°79’). The soil in these regions was yellow brown soil. xq (the detailed physical and chemical properties are shown in **Supplementary Table S2**). All areas adopt the same fertilization management measures. The base fertilizer application amount was compound fertilizer (NPK: 10:10:25) 35 kg/acre, fermented oil meal 30 kg/acre, calcium magnesium phosphate 25 kg/acre, and it was uniformly and evenly applied in the fertilizer furrow before ridge formation. Subsequent fertilization: 1% and 5% compound fertilizer (nitrogen-phosphorus-potassium ratio: 10:10:25) were used for topdressing on the day of transplanting and the seventh day, respectively, and the remaining topdressing was completed within 25–30 days after transplanting. Three separate tobacco leaf samples were collected from each region, with the sampling method being independent. Each area collected 3 biological replicate samples, each replicate sample containing 20 tobacco leaves that met the specified location and size standards. All target leaves were marked before harvest. A total of 18 replicate samples were collected. The samples from each region were divided into two groups, where one group was used for sequencing of the inter-leaf microbial community [including 16S rRNA gene sequencing (16S) and internal transcribed spacer (ITS)], and the other group was used for conventional chemical component analysis.

### 2.2 Determination of the conventional chemical components of leaves

The collected samples were ground into powder. Approximately 0.1 grams of the sample was added to 1 milliliter of 80% ethanol for thorough mixing. The sample was extracted in an 80°C water bath for 30 minutes, then centrifuged at 3000 revolutions per minute at room temperature for 5 minutes. The starch content was determined using the Sol biological assay kit (Beijing, China). 0.25 grams of the sample was weighed and placed in a 50 mL bottle. The powder was mixed with 25 mL of 5% acetic acid by shaking for 30 minutes and then filtered through a membrane filter. The filtrate was subjected to flow analysis to determine the total sugar, reducing sugar, total nitrogen, total plant alkaloids, potassium, and chlorine contents (Zhao et al., 2022). The ratios of disaccharides, nitrogen to alkaloids, and sugar to alkaloids were also calculated.

### 2.3 Microbial elution and enrichment from tobacco leaf surfaces

Using sterilized scissors, cut a portion of the tobacco leaves into fragments approximately 5 cm^2^ in size, avoiding the veins. Weigh 20 grams of the sample and place the tobacco leaf fragments in a sterilized conical flask. Add 250 ml of 1% sterile PBS buffer solution. Shake the conical flask with a shaker and perform centrifugation to collect the microorganisms on the leaves. Wash the precipitate, centrifuge again and discard the supernatant. Place the coarse precipitate in a -80°C refrigerator for storage, so that the subsequent sequencing work can proceed normally (Ding et al., 2023).

### 2.4 Extraction of DNA from tobacco leaves and Illumina Mi-Seq sequencing

Genomic DNA was extracted using the SDS method. The purity and concentration of the DNA were detected by agarose gel electrophoresis. A PCR reaction system was prepared using 30 ng of high-quality genomic DNA sample and corresponding fusion primers (Ding et al., 2023). The bacterial 16S rRNA gene was amplified using the primers 799F-1193R (AACMGGATTAGATACCCKG-ACGTCATCCCCACCTTCC) (Anguita-Maeso et al., 2022). The PCR program was as follows: 98\u00B0C for 1 min, followed by 30 cycles of 98\u00B0C for 10 s, 50\u00B0C for 30 s and 72\u00B0C for 30 s, and a final extension at 72\u00B0C for 5 min. PCR products were detected by electrophoresis with 2% agarose gel. The samples were mixed in equal amounts according to the concentration of PCR products. After full mixing, PCR products were detected by 2% agarose gel electrophoresis. The PCR amplification products were purified using Agencourt AMPure XP magnetic beads and dissolved in Elution Buffer, labeled, and the library was constructed (Hasrat et al., 2021). The fragment range and concentration of the library were detected using the Agilent 2100 Bioanalyzer (Becker et al., 2010). The qualified libraries were sequenced using the sequencer according to the insert fragment size. The ITS gene of fungi was amplified using the ITS1F primer (Adams et al., 2013), with the exception of the annealing temperature of 55°C and the number of cycles of 35 times, the PCR conditions were the same as those for the 16S rRNA gene gene (Sun et al., 2024).

### 2.5 Analysis of microbial amplification sequences of tobacco leaves

The original sequencing data was processed as follows: Using the software cutadapt v2.6, the primers and adapter contaminants were removed. A window length of 30 bp was set. If the average quality value of the window was lower than 20, the end sequence of the read was cut off from the window and reads with a final length lower than 75% of the original read length were removed. Reads containing N and those with low complexity (consecutive 10 ATCG) were also removed to obtain the final clean data. After removing the Barcode and primer sequences from the downstream data, FLASH (Fast Length Adjustment of Short reads, v1.2.11) efficiently assembled the read segments into long sequences based on the overlapping regions (Magoč and Salzberg, 2011). This algorithm set the minimum matching length to 15 bp and allowed a mismatch rate of 0.1. Using the software USEARCH (v7.0.1090_i86linux32) under a 97% similarity threshold, OTUs were generated. Subsequently (Edgar, 2013), the UCHIME v4.2.40 software was used to remove potential chimeric sequences generated by PCR amplification. The method of comparing with the 16S chimeric database: gold database (v20110519) and the ITS chimeric database: UNITE (v201706 28) was adopted to remove chimeras. All processed tags were compared with the OTU representative sequences using usearch_global to generate a comprehensive OTU relative abundance statistics table (Edgar et al., 2011). By setting the confidence threshold to 0.6, the classification affiliation of each OTU was clearly defined, and the OTU representative sequences were compared with the corresponding databases [16S using RDP (Cole et al., 2009), ITS using UNITE (Nilsson et al., 2019b)]. Finally, all OTUs that could not be successfully annotated were deleted.

### 2.6 Data processing

The RDA and NMDS plots were generated using R software (version 4.4.1, developed by the R Statistical Computing Foundation in Vienna, Austria) to visualize the beta diversity. Bar charts were created using the ggplot2 package (v3.5.1). The differences in chemical components within each region were evaluated by calculating the standard deviation (SD) between three biological replicates. The results were presented in the form of mean ± standard deviation in tables and graphs to illustrate the dispersion of the data. Statistical analysis between fresh leaves and processed leaves was conducted using the independent sample t-test. The statistical analysis method for significant differences between regions was as follows: Based on the results of Duncan’s *post hoc* test, groups with no significant differences are marked with the same letter (p<0.05). The co-occurrence network analysis was performed using the WGCNA package (Langfelder and Horvath, 2008), and the microbial co-occurrence network was constructed based on the Spearman correlation coefficient at the OTU level. Only the relationships with absolute values ≥ 0.6 and p-values ≤ 0.001 after Benjamini & Hochberg (BH) correction were retained to define the edges in the network. Visual analysis was conducted using the Gephi software (Bastian et al., 2009). Variance decomposition analysis (VPA) using the ‘vegan’ package was performed to detect the response of microbial communities to environmental factors (Oksanen et al., 2022). PICRUSt2 (version 2.5.1) was used to predict the functional potential of the bacterial community (Langille et al., 2013; Wright and Langille, 2025).

## 3 Results and analysis

### 3.1 Environmental parameters and chemical composition of the blades

The climate data indicate that there are significant spatial distribution differences in the climate parameters of each sampling area (**Supplementary Table S1**). The SC area is a cold and humid region, with its average temperature, effective accumulated temperature, and sunshine duration all being lower than those of other areas, but the precipitation reaches 198.6 millimeters. The sunshine duration in DJ and FQ areas is higher than that of other areas, but the precipitation is less, being 121.8 millimeters and 129.3 millimeters respectively. The precipitation extreme value in FG area is 227.00 millimeters, while the precipitation in HP area is only 53.77 millimeters, belonging to a drought area. The XF area shows transitional climate characteristics, with its sunshine duration and precipitation both being at the intermediate gradient (**Supplementary Table S1**).

**Figure 1:**
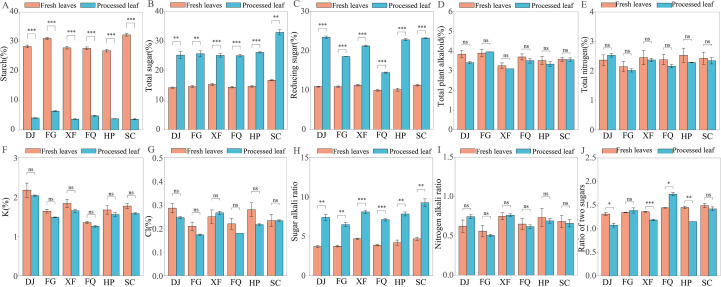
Comparison of chemical composition contents of fresh plant leaves and processed leaf samples among different regions. **(A)** starch, **(B)** represents total sugar, **(C)** represents reducing sugar, **(D)** represents total plant alkaloids, **(E)** represents total nitrogen, **(F)** represents potassium, **(G)** represents chlorine, **(H)** represents sugar alkaloid ratio, **(I)** represents nitrogen-to-alkaloid ratio, **(J)** represents ratio of two sugars. Red indicates fresh leaves, blue indicates processed leaf slices. The “***” symbol indicates p < 0.001, “**” indicates p < 0.01, “*” indicates p < 0.05, “ns” indicates p > 0.05.

The analysis of the chemical components of the leaves revealed significant changes during the processing (**Figure 1**). Specifically, the ratios of starch, total sugar, reducing sugar, and the ratio of two sugars all showed significant changes before and after processing (p < 0.05, **Figures 1A–C, J**). Among them, the starch, total sugar, and reducing sugar contents of fresh tobacco leaves were 26.87% - 32.25%, 14.24% - 16.74%, and 9.96% - 11.26% respectively, while the starch, total sugar, and reducing sugar contents of processed tobacco leaves were 3.67% - 6.41%, 25.06% - 32.96%, and 14.15% - 23.47% (**Figures 1A–C**). There were no significant differences in total alkaloids, total nitrogen, potassium, and chlorine before and after processing (p > 0.05, **Figures 1D–G**). From the perspective of regional differences, in the fresh leaf samples, the starch content in the SC area was the highest, followed by the FG area. Meanwhile, the total sugar content in the SC area was significantly higher than that in other areas (**Supplementary Table S3**, **Figure 1A**). After processing, the starch decomposition degree in the FG area was the lowest, followed by the FQ area, while the starch decomposition degree in other areas was higher (**Supplementary Table S4**, **Figure 1A**).

### 3.2 Composition, interactions and diversity of bacterial and fungal communities

**Figure 2:**
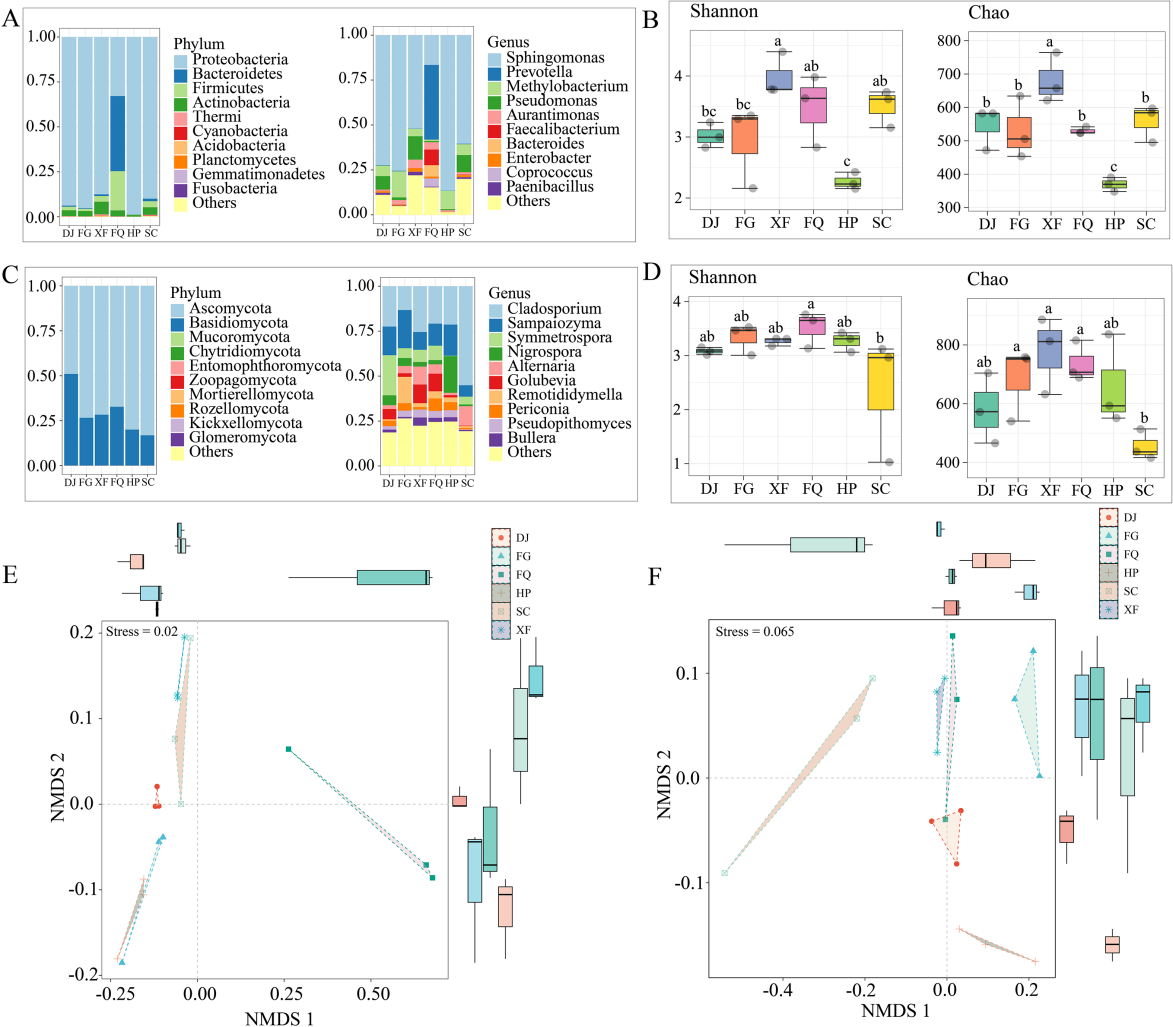
Analysis of diversity and community structure of bacterial and fungal communities between regions. **(A)** Stacked plot of species abundance at the 16sRNA phylum and genus levels. **(B)** α-diversity analysis of 16sRNA. **(C)** Stacked plot of species abundance at the ITS phylum and genus levels. **(D)** α-diversity analysis of ITS. **(E)** NMDS analysis of 16sRNA. **(F)** NMDS analysis of ITS.

To investigate the spatial heterogeneity of microbial communities in different regions, the relative abundances of bacterial and fungal communities at the phylum and genus levels were analyzed. The results showed that there were significant differences in the composition of microbial communities in different regions. In the bacterial community, Proteobacteria (32.81%- 98.58%) was the dominant group, and the relative abundances of Bacteroidetes (41.86%) and Firmicutes (21.79%) in the FQ samples significantly increased. At the genus level, *Sphingomonas* (16.56%- 86.50%), *Methylobacterium* (4.16%- 14.56%), and *Pseudomonas* (0.53%- 16.56%) were the common dominant genera in all regions, while the relative abundance of *Prevotella* (41.58%) in the FQ sample was significantly higher than that of the *Pseudomonas* (0.53%) genus (**Figure 2A**). The fungal community was dominated by Ascomycota, followed by Basidiomycota. At the genus level, *Cladosporium* and *Sampaiozyma* dominated the FG, XF, and FQ samples (**Figure 2C**).

The Alpha diversity analysis results showed that the bacterial Shannon diversity index of the XF sample was significantly higher than that of the other samples, while the diversity of the HP sample was the lowest (**Figure 2B**). The Shannon diversity index of the fungal community was only significantly lower than that of the FQ sample in the SC sample (**Figure 2D**). In terms of species richness, the bacterial Chao1 index of the XF sample was significantly higher than that of the other samples, and the HP sample was the lowest (**Figure 2B**); the Chao1 index of the fungal community was significantly lower than that of the FQ sample in the SC sample (**Figure 2D**). Through non-metric multidimensional scaling analysis (NMDS), it was shown that the bacterial and fungal communities from different regions could be significantly classified into three main clusters (stress ≤ 0.2, **Figures 2E, F**), indicating that the microbial community structure has significant spatial heterogeneity.

**Figure 3:**
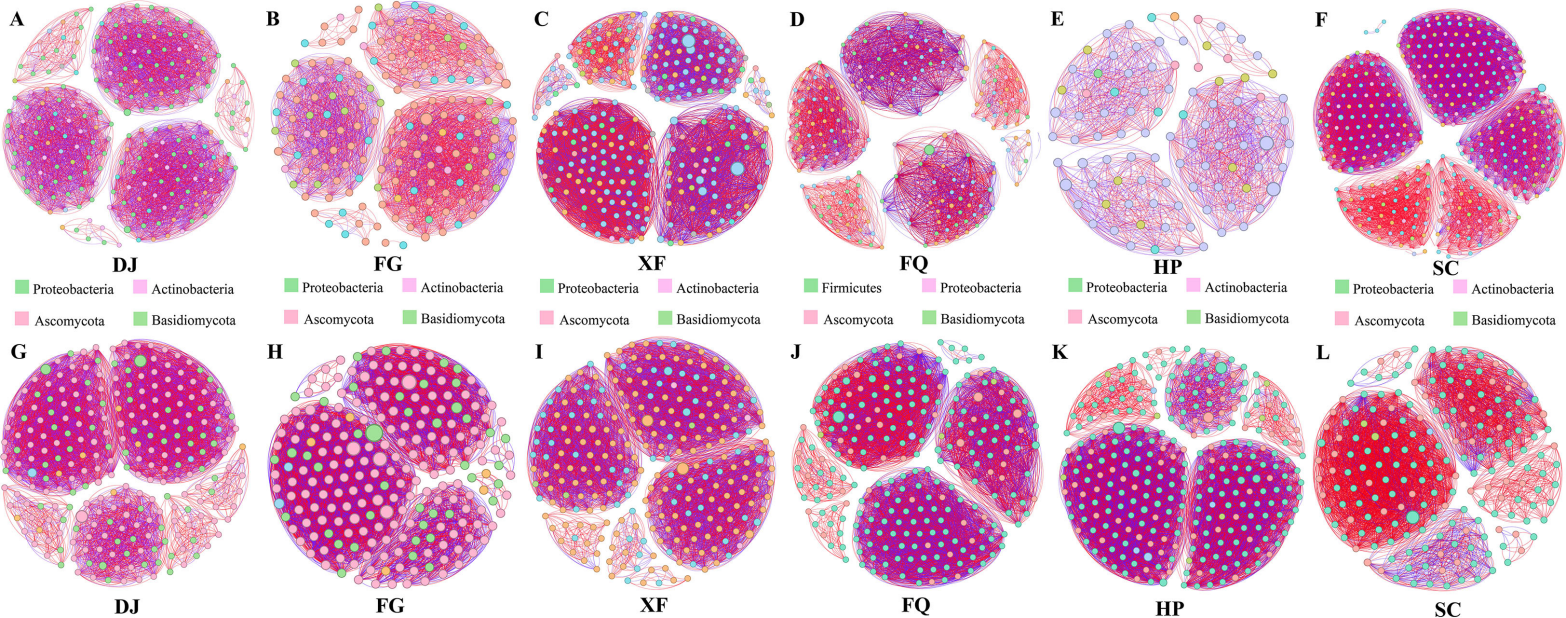
Co-linear network analysis of bacterial and fungal communities across regions. **(A–F)**, Network diagrams of OTU-level microbial communities in 16sRNA samples from different regions. **(G–L)**, Network diagrams of OTU-level microbial communities in ITS samples from different regions. The color of the points represents different phyla, and the lines between the points represent the correlations between OTUs. Red lines indicate positive correlations. Blue lines indicate negative correlations.

Based on OTU for co-occurrence network analysis, the interaction characteristics of bacterial and fungal communities between regions were explored. The bacterial community analysis showed that the samples of XF, FQ, and SC had similar numbers of nodes (381, 339, and 361 respectively), while the sample of HP had the lowest number of nodes (93) (**Supplementary Table S5**; **Figures 3C–F**). Compared with the HP sample, the edge numbers of GJ, FG, XF, FQ, and SC samples were 4.84, 3.0, 16.8, 12.3, and 12.4 times higher, respectively, and the positive correlation ratios were all relatively high. Among them, the positive correlation ratios of FQ (60.09%) and SC (60.99%) samples were similar. Particularly noteworthy was that the FG sample, although having relatively lower edge numbers (3472) and node numbers (159), had the highest positive correlation ratio (66.16%) (**Supplementary Table S5**; **Figures 3A–F**). The fungal community analysis results showed that the sample of XF had the most edge numbers (18861) and node numbers (381), while the sample of SC had the lowest edge numbers (4461) and node numbers (184) (**Supplementary Table S5**; **Figures 3I, M**). The positive correlation ratios of DJ, FG, FQ, and HP samples were similar, being 51.88%, 52.52%, 54.95%, and 53.18% respectively (**Supplementary Table S5**; **Figures 3G, H, K, L**), while the positive correlation ratio of SC sample was the highest (85.99%) (**Supplementary Table S5**; **Figure 3M**). Additionally, the edge numbers of DJ (7462) and FG (7116) samples were similar, and the edge numbers of FQ (11433) and HP (10194) samples were also relatively close (**Supplementary Table S5**; **Figures 3G, H**).

### 3.3 Correlation between environmental parameters and bacterial and fungal communities

**Figure 4:**
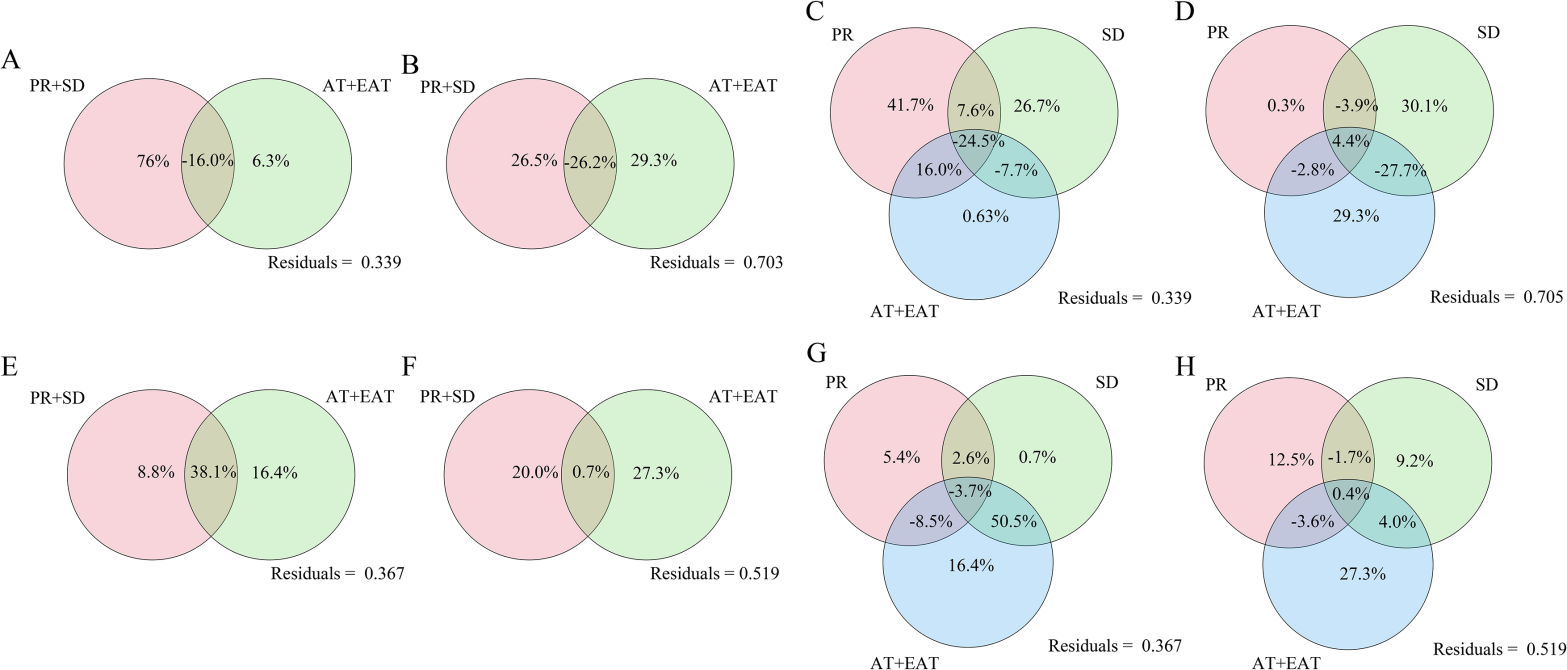
Variance partitioning analysis (VPA) of the effects of different climatic factors on the community structure of bacteria and fungi. The effects of different climatic factors on bacterial community samples at the phylum level **(A, C)** and genus level **(B, D)**. The effects of different climatic factors on fungal community samples at the phylum level **(E, G)** and genus level **(F, H)**. Here, PR: precipitation; SD: sunshine duration; AT: average temperature; EAT: effective accumulated temperature.

The influence of climatic factors on the microbial community was evaluated through Variance Partitioning Analysis (VPA). The results showed that the combination of PR + SD had the most significant effect on the bacterial community at the phylum level, explaining 76% (**Figure 4A**), while AT + EAT explained 51.5% of the variation at the genus level (**Figure 4B**). For the fungal community, the combination of AT + GDD was the main driving factor, explaining 14.6% and 27.3% of the variation at the phylum and genus levels respectively (**Figures 4B, C**). Additionally, PR had the greatest contribution to the bacterial phylum level (41.7%), followed by SD (26.7%). SD showed the highest degree of explanation at the genus level (**Figures 4C, D**). Temperature was the main driving factor for fungi, showing the highest influence at both the phylum and genus levels. SD and AT + EAT had a synergistic effect. Overall, the explanatory degrees of temperature and sunshine duration for the fungal community were relatively low (**Figures 4E, H**).

**Figure 5:**
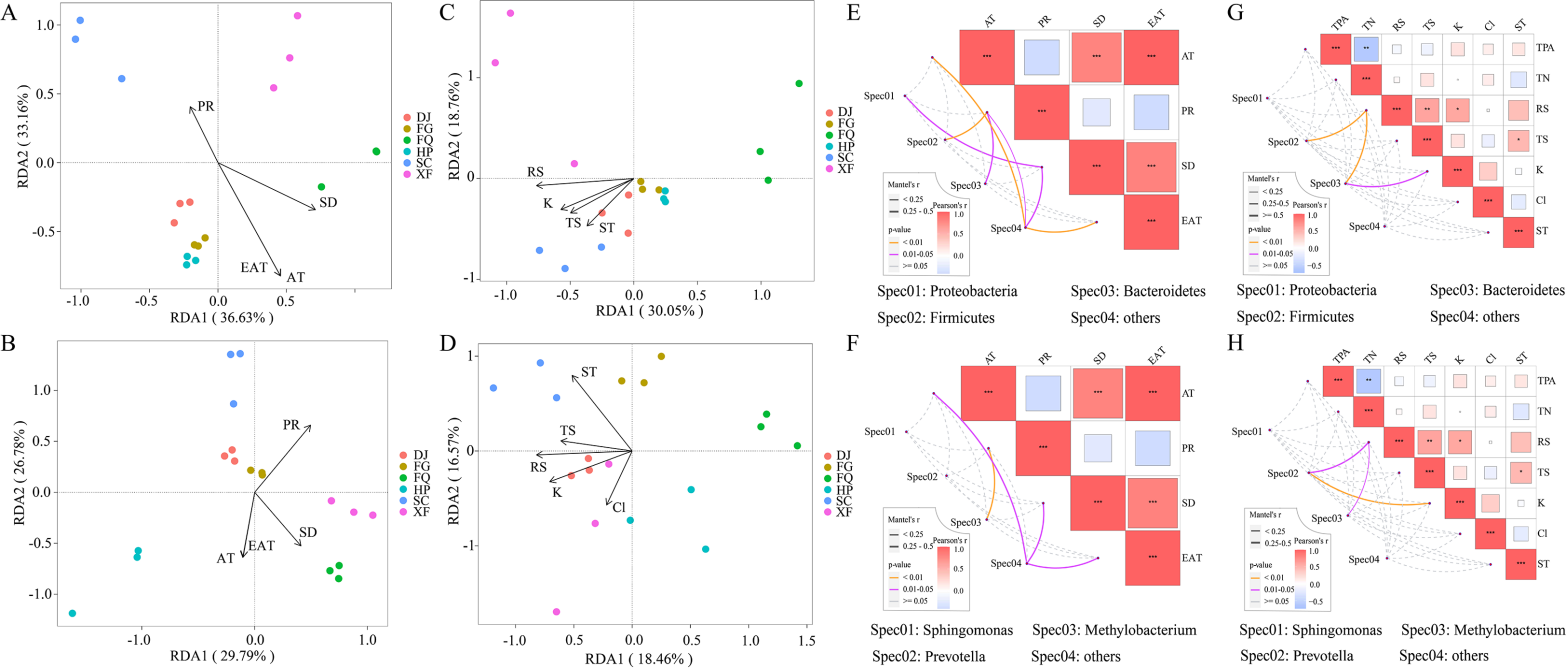
The influence of climatic factors on microbial communities. Redundancy analysis (RDA) of 16sRNA **(A, C)** and ITS **(B, D)** samples from different regions and environmental factors, as well as chemical components of fresh leaves at the OUT level. Mantel test of samples at the phylum level (**E, G**) and genus level (**F, H**) from different regions. The top three phyla or classes are combined into Spec01, Spec02, and Spec03. The color and thickness of the lines represent the P and R values of the Mantel test, where “***” indicates p < 0.001, “**” indicates p < 0.01, and “*” indicates p < 0.05.

The RDA shows that the correlations between environmental factors and bacterial and fungal communities are similar in regional terms. PR is the main environmental factor influencing the bacterial and fungal communities in samples from the SC and XF zones. AT, EAT, and SD have similar effects and jointly influence the microbial communities in samples such as HP and FQ (**Figures 5A, B**). Additionally, AT and EAT are the main drivers for bacteria (r^2^ > 0.8, **Supplementary Table S6**), while SD and RS act as secondary influencing factors. ST has the strongest explanatory power for fungi (r^2^ > 0.840, **Supplementary Table S6**), and PR and RS also have significant effects on fungi (r^2^ > 0.6, **Supplementary Table S6**). Specifically, bacteria are more dependent on temperature effects, while fungi are more strongly regulated by PR and ST (**Figures 5A–D**).

The correlation between microbial communities and climatic factors, as well as physicochemical factors of fresh leaves, was analyzed through Mantel tests. The results show that AT, SD, and EAT are significantly positively correlated, while PR is negatively correlated with all three. *Proteobacteria*, *Firmicutes*, and *Bacteroidetes* are significantly positively correlated with climatic factors. Among them, PR significantly affects *Firmicutes*, *Bacteroidetes*, and *Methylobacterium*, while *Proteobacteria* mainly responds to SD (**Figures 5E, F**). *Firmicutes*, *Bacteroidetes*, *Methylobacterium*, and *Prevotella* are significantly correlated with reducing sugar and potassium (**Figures 5G, H**).

### 3.4 Functional forecast analysis

**Figure 6:**
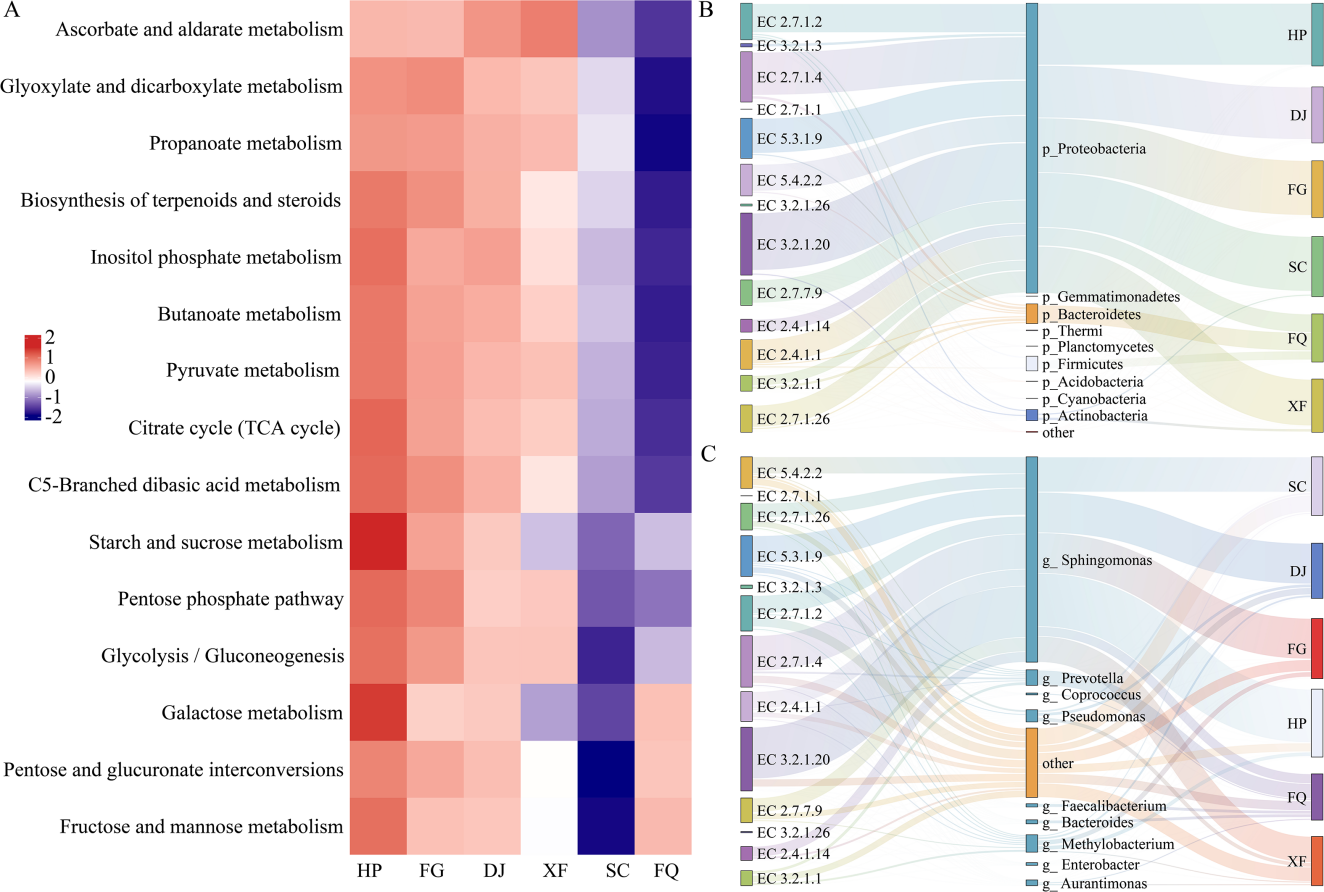
Functional predictions of bacterial communities in different regions using PICRUSt2. **(A)** Functional predictions of carbohydrates in samples from different regions. **(B)** Sankey Diagram showing enzymes related to starch and sucrose metabolism pathways at the phylum level. **(C)** Sankey Diagram showing enzymes related to starch and sucrose metabolism pathways at the genus level.

Based on PICRUSt2, the functional predictions of bacterial communities in six regions were conducted, and the expression patterns of genes related to carbohydrate metabolism pathways were analyzed through heatmap clustering. The results showed that the FQ samples exhibited higher gene abundances in the galactose metabolism, pentose and glucuronic acid interconversion, and fructose and mannose metabolism pathways. The gene abundances of the remaining metabolic pathways in FQ and the SC samples were significantly lower than those of the HP, FG, DJ, and XF region samples (**Figure 6A**). Notably, there were significant gene expression differences in the fruit sugar and mannose metabolism, starch and sucrose metabolism, and galactose metabolism pathways among the six regions (**Figure 6A**).

The contribution of bacterial communities in different regions to the starch and sucrose metabolism pathways was evaluated at the phylum and genus levels using Sankey Diagram. The results indicated that *Proteobacteria*, *Firmicutes*, *Bacteroidetes*, and *Actinobacteria* were the main sources of related enzymes (**Figure 6B**). Among them, the 1,4-α-glucan branching enzyme (EC 2.4.1.14) was almost entirely derived from *Proteobacteria* (**Figure 6B**). Additionally, *Bacteroidetes* had a significant contribution to hexokinase (EC 2.7.1.1), while *Firmicutes* was the main source of β-fructofuranosidase (EC 3.2.1.26) (**Figure 6B**). At the genus level, *Sphingomonas*, *Pseudomonas*, *Prevotella*, and *Methylobacterium* were the main sources of most enzymes, with α-amylase (EC 3.2.1.1), phosphoglucomutase (EC 5.4.2.2) etc, mainly contributed by Other (**Figure 6C**).

### 3.5 Metabolic reconstruction of sucrose and starch pathways

**Figure 7:**
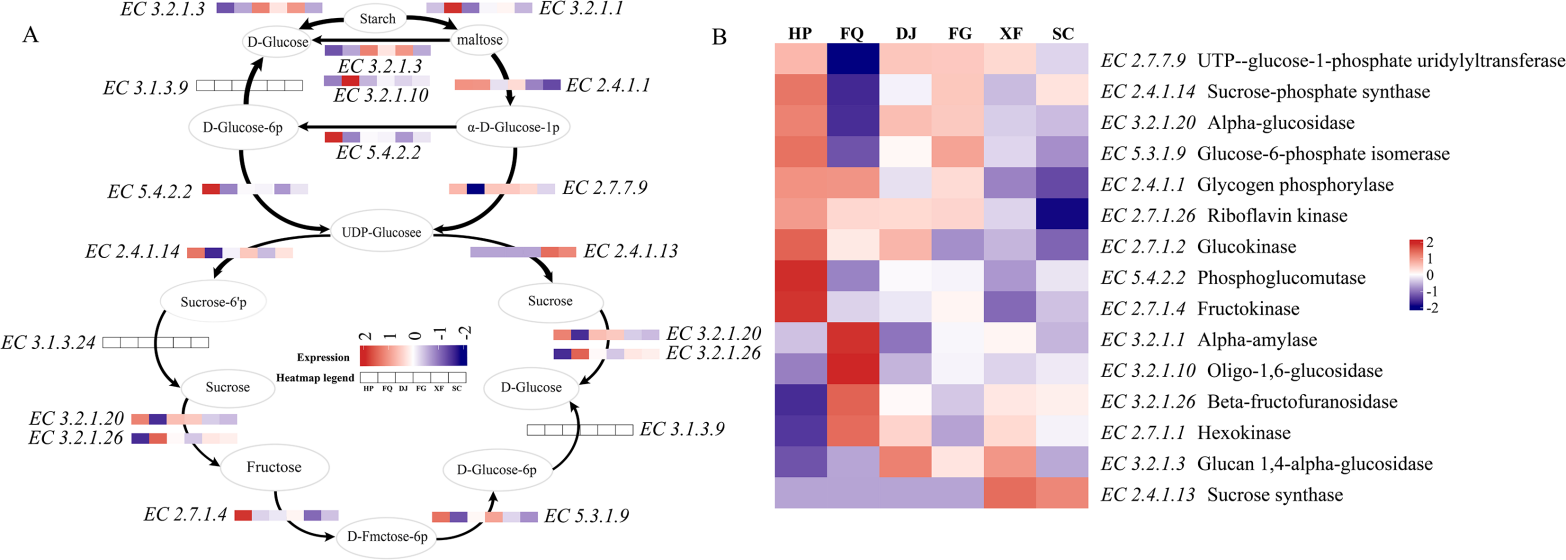
Reconfiguration of metabolic pathways for sucrose and starch metabolism. **(A)** Starch and sucrose metabolism in samples from different regions. **(B)** Heat map used to analyze the differences in starch and sucrose metabolism-related enzyme genes in samples from different regions.

Based on the predicted abundance of genes encoding key enzymes, we reconstructed the putative metabolic pathways for starch and sucrose in the phyllosphere microbial community (**Figure 7**). Starch can be metabolized through two potential pathways: the direct pathway and the indirect pathway. In the direct pathway, genetic potential existed for the direct conversion of starch to D-glucose. This process appeared to be particularly prominent in the DJ and XF samples, as indicated by their higher predicted abundance of genes encoding Glucan 1,4-alpha-glucosidase (EC 3.2.1.3) (**Figures 7A, B**). In the indirect pathway, the model predicts that starch could be initially catalyzed by Alpha-amylase (EC 3.2.1.1) to generate maltose. Maltose may then be hydrolyzed into D-glucose and α-D-glucose-1-phosphate by the coordinated action of several enzymes. During this process, Glucan 1,4-alpha-glucosidase (EC 3.2.1.3) has advantages in the expression of DJ and XF samples, while Oligo-1,6-glucosidase (EC 3.2.1.10) has the most significant expression in the FQ region (**Figures 7A, B**). The metabolic fate of α-D-glucose-1-phosphate may proceed through two routes. The first route, involving its conversion to D-glucose by Glucose-6-phosphatase (EC 3.1.3.9), appears less likely, as genes encoding this enzyme were not detected in any samples. The second route, mediated by Phosphoglucomutase (EC 5.4.2.2) and UTP-glucose-1-phosphate uridylyl transferase (EC 2.7.7.9), seems to be the dominant potential pathway. It is noteworthy that the XF and SC samples exhibited a significantly higher predicted abundance of genes encoding Sucrose synthase (EC 2.4.1.13), which is predicted to drive the conversion of UDP-glucose to sucrose. The absence of detected genes for Sucrose-phosphate synthase (EC 2.4.1.14) further suggests that sucrose synthesis in this community may rely primarily on the Sucrose synthase pathway (**Figures 7A, B**). Overall, the functional profile indicates a heightened genetic potential for starch and sucrose catabolism in FQ samples, inferred from the highest predicted gene abundances for Alpha-amylase and Oligo-1,6-glucosidase. The gene abundance for Beta-fructofuranosidase (EC 3.2.1.26), which may promote sucrose hydrolysis, was also far exceeding other samples. The XF and SC samples appear to be primarily associated with UDP-glucose generation and sucrose synthesis, facilitated by their high expression of Sucrose synthase. Meanwhile, HP, DJ and FG samples play a role in the phosphorylation and isomerization processes, as suggested by the active expression of Fructokinase (EC 2.7.1.4) and Glucose-6-phosphate isomerase (EC 5.3.1.9), potentially facilitating the conversion of fructose into D-glucose (**Figures 7A, B**).

## 4 Discussion

### 4.1 Differential responses of the microbial communities at the leaf level to regional climatic factors

To study the climate adaptability and functional changes of the microbial communities in the periphery of tobacco leaves, this study used high-throughput sequencing technology to analyze the changes in the microbial communities in six tobacco-growing areas, revealing the significant influence of climatic factors on the composition of microorganisms and exploring the functional changes of the communities. The results showed that precipitation was the most critical factor driving the structure of the bacterial community (VPA explained 41.7%), while the duration of sunlight and temperature had a more significant impact on the fungal community (**Figures 4G, H**). This finding is consistent with previous studies, such as the significant decrease in the diversity of leaf-edge bacteria in Huangjiao leaves under reduced precipitation (Wang et al., 2023), and the increase in the abundance of pathogenic fungi in the leaf-edge fungal community under long-term warming conditions in Yang leaves (Bálint et al., 2015). However, the response of the fungal community to precipitation and sunlight duration was less pronounced in this study, which may be related to the unique microenvironment of tobacco leaf edges (such as the thickness of the leaf cuticle and the composition of volatile organic compounds) buffering the direct effects of climate (Huang et al., 2024). In the bacterial community, the *Proteobacteria* as the core group, its abundance was significantly positively correlated with precipitation (p < 0.01; **Figure 5E**). This finding is consistent with the pattern reported for *Pseudomonadaceae* in the phyllosphere of cucumbers and tomatoes, where an increase in their abundance is observed following precipitation (Allard et al., 2020). However, in the specific ecological niche of tobacco leaves, we believe that the emergence of the dominant bacterial community is not only due to the availability of water, but also related to the cooperative strategies they share under humid conditions. The high-humidity leaf surface environment may facilitate the diffusion and sharing of “public goods” such as extracellular polysaccharides and iron carriers, enabling the Proteobacteria groups (such as *Pseudomonas*) that are good at producing these substances to establish mutually beneficial networks and jointly resist competition from other microorganisms or pathogen invasion, thereby occupying a dominant position in the community (Pantigoso et al., 2025). This study also found that the *Firmicutes* and *Actinobacteria* were significantly enriched in the arid area(**Supplementary Table S1**, **Figure 2A**), which is consistent with the drought-resistant mechanism of *Streptomyces* reported by D. Metze et al (Metze et al., 2023). This may occur because high environmental stress selectively eliminates low-abundance taxa while retaining more drought-resistant groups within the community (Lynch and Neufeld, 2015; Wang et al., 2018). We speculate that under the drought stress in tobacco leaves, these two groups may have adopted complementary survival strategies. The Firmicutes phylum, due to its ability to form spores, might enter a dormant state under extreme drought and high temperature radiation (Aydogan et al., 2018). The Actinobacteria phylum, on the other hand, may utilize its more complex secondary metabolic pathways to produce extracellular polysaccharides with moisturizing properties or antibacterial substances. These substances can form physical biofilms on the leaf surface, which not only create a micro-humid environment for the bacteria themselves but may also indirectly reduce water evaporation from the host leaves (Jordaan et al., 2020). It is worth noting that the long duration of high sunlight and low precipitation in the FQ zone led to the dominance of *Bacteroidetes* and *Firmicutes* as the dominant groups (**Supplementary Table S1**, **Figure 2A**). This phenomenon suggests that under the dual stress of strong light and drought, the microbial community on tobacco leaves may have undergone a functional transformation of “metabolic synergy”. The Bacteroidetes phylum is known for its ability to degrade complex polysaccharides, and may be responsible for decomposing the damaged plant cell walls or secretions caused by light stress (Zeng et al., 2025), while the Firmicutes phylum is good at fermenting the resulting simple sugars. This “decomposition-fermentation” metabolic coupling not only efficiently utilizes limited resources, but also may produce metabolites such as short-chain fatty acids, which can feedback regulate the physiological state of the leaves or inhibit pathogens, thereby helping the entire system adapt to the stressful environment (Zou et al., 2024). In addition, the study found that the interaction of climatic factors has regional specificity on the impact of microbial communities. For example, the bacterial community in high precipitation areas is dominated by Pseudomonas, while the fungal community is dominated by *Cladosporium* (**Supplementary Table S1**, **Figure 2C**), which is partially consistent with the enrichment pattern of *Methylobacterium* in citrus leaf interstitial environments under humid conditions (Yuan et al., 2025). In low precipitation areas, *Nigrospora* significantly increases in the fungal community (**Supplementary Table S1**, **Figure 2C**), which is consistent with the general rule that drought promotes the prevalence of pathogenic fungi (Malik and Bouskill, 2022), but differs from the conclusion of Bálintet al. regarding the increase of pathogenic fungi in poplar under high temperatures (Bálint et al., 2015). This prevalence might be because ascomycete groups have multiple strategies to cope with drought stress, such as the production of specific proteins, modification of cell membranes, slow growth rate, or even dormancy (Shu and Huang, 2022).

### 4.2 Functional adaptability of phyllosphere microorganisms under climate influence and its impact on host metabolism

The functional prediction of microbial communities (PICRUSt2) indicates that climate factors indirectly affect the sugar metabolism of flue-cured tobacco by regulating the distribution of key enzymes in the carbon metabolism pathways (**Figures 6**, **7**). For instance, in regions with high sunlight and low precipitation, the bacterial community has the highest abundance of genes for galactose metabolism and pentose conversion pathways (**Supplementary Table S1**, **Figure 6A**), which is associated with the enrichment of the *Firmicutes*(**Figure 2A**). This result is similar to that of Yuan et al., where summer high-temperature weather parameters have a significant impact on amino acids and sugars in leaves, and citrus plants exhibit vigorous vegetative growth (Yuan et al., 2025). Additionally, the *Proteobacteria* serves as the main source of sucrose synthase (EC 2.4.1.13), promoting sucrose synthesis. The *Firmicutes* promotes sucrose hydrolysis by secreting β-fructofuranosidase (EC 3.2.1.26) (**Figure 6B**), thereby helping the host cope with water stress. This finding is similar to the osmotic sensing and osmotic regulation mechanism proposed by Malik, Roy Chowdhury et al., where microbial communities produce non-structural carbohydrates and amino acids under drought conditions, increasing the quantity and quality of plant organic matter and enabling plants to adapt to the negative impacts of drought (Roy Chowdhury et al., 2019; Malik et al., 2020).

Recent studies have demonstrated that *Proteobacteria* can express genes for enzymes such as 1,4-α-glucan branching enzyme (EC 2.4.1.14), phosphogluconate dehydrogenase (EC 5.4.2.2), and sucrose synthase (EC 2.4.1.13), promoting the growth of Japanese japonica rice under drought stress (Chen et al., 2025), which is consistent with the results of this study (**Figures 2A**, **6B**, **7B**). It is notable that these enzymes mainly originate from the *Proteobacteria*(**Figure 6B**), which is consistent with the function of rhizobia in the core microbiome of the yellow poplar, indicating the conservation across host species (Wang et al., 2023). Although the SC region has a relatively high total sugar and reducing sugar content, the expression of the starch and sucrose metabolic pathways is relatively low. On one hand, this may be related to the higher initial substrate starch content (**Figure 1A**). On the other hand, it may be due to plants often accumulate soluble sugars as osmotic protectants under low temperatures, and the weakened respiration inhibits the activity of metabolic enzymes, preventing them from being rapidly utilized (Shu et al., 2009; Wang et al., 2020). Further analysis of the starch and sucrose metabolic pathways revealed that no Glucose-6-phosphatase (EC 3.1.3.9) gene expression was detected in the microbiota, and the generation of D-glucose mainly relies on the degradation pathways of starch and sucrose (**Figure 7**). This may be because the microbiota is more likely to utilize Glucose-6-phosphatase directly for glycolysis rather than converting it into free glucose for host utilization (Khanna et al., 2014). It is noteworthy that studies have shown that key enzyme genes have regional metabolic characteristics (**Figure 7B**), which is consistent with previous research. The microbial community may undergo significant functional changes in response to one or more climate factors (Compant et al., 2010; Singh et al., 2018; Wang et al., 2023), such as the increased expression levels of drought-resistant genes like sucrose synthase and glucose-6-phosphate isomerase in roots under drought and high-temperature conditions (Chen et al., 2025). Specifically, in arid regions, microbial communities showed a higher predicted abundance of genes related to starch degradation (such as α-amylase) (**Figure 7**). This is consistent with the observed enrichment of Firmicutes/Actinobacteria and leads to a key hypothesis: under drought stress, the microbial communities in tobacco leaves may shift from utilizing easily accessible photosynthetic products (such as sucrose) from the host to exploring more stable storage carbon sources - starch. This metabolic strategy shift may, on one hand, alleviate the carbon limitation imposed on the microbes by the reduced export of photosynthetic products (sucrose) due to stomatal closure, on the other hand, the glucose produced by degrading starch may be partially used by the microbes for synthesizing compatible solutes (such as trehalose, proline), which may also seep out and help the host cells maintain osmotic balance (Zeng et al., 2025). On the contrary, in areas with high precipitation, the predicted abundance of genes related to sucrose synthesis (such as sucrose synthase) was higher (**Figure 7**). We hypothesize that this reflects a “growth-oriented” metabolic division of labor between the leaf-associated microbial community and the host under conditions of abundant water and vigorous photosynthesis. Abundant precipitation may wash away some leaf-associated microorganisms, but it also brings new nutrients. Dominant groups such as Proteobacteria may actively participate and even modulate the conversion and distribution of photosynthetic products in leaves towards sucrose, thereby promoting the growth of the host and obtaining stable carbon flow (Chen et al., 2025).

Furthermore, our functional prediction indicates that key enzyme functions, such as α-amylase, are mainly attributed to the classification group labeled as “Other” (**Figure 6C**). This limits our ability to determine the exact contributors of the classification groups, but this observation itself has significant ecological significance. It is consistent with the concept of the rare biosphere, which states that in microbial ecosystems, key functions are often encoded by low-abundance classification groups (Jousset et al., 2017). These rare classification groups may act as functional experts, maintaining key metabolic pathways without bearing the pressure of high-density populations, thereby enhancing the functional recovery ability of the community under stress (Banerjee et al., 2018). Based on this, we propose a working hypothesis for future validation: when facing drought or temperature fluctuations, the tobacco phyllosphere microbial community may enhance its functional redundancy by retaining core metabolic functions, such as starch degradation, within highly diverse rare taxa. If this mechanism holds true, these rare taxa could provide a potential ecological safeguard for the host under environmental disturbances. Therefore, future studies should integrate long-term spatiotemporal observations, metagenomic/metatranscriptomic approaches, and microbial culture-based methods to test this hypothesis.

### 4.3 The ecological adaptability of microbial communities to climatic factors

Climate factors can alter the relative abundance of these key microorganisms, thereby strongly influencing the overall structure of the phyllosphere community (Aydogan et al., 2018). In this study, the main network hubs, as well as the taxonomic groups adjacent to the network hubs, were significantly correlated with temperature and precipitation variables, indicating that the transfer of key species may disrupt the stability of the entire microbial community in the leaf ecosystem as spatial or temporal conditions change. The co-occurrence network analysis revealed that the network in the high-temperature and low-humidity area was simpler compared to other areas (**Figure 3E**), possibly due to extreme drought filtering out low-tolerance groups and retaining only a few drought-resistant bacteria. For example, in arid environments, microorganisms exhibit lower species richness, high abundance of a few dominant species, and relatively lower complexity of the microbial community (Chen et al., 2022; Shu and Huang, 2022). Additionally, an increase in temperature stimulates various biological interactions, such as predation, parasitism, competition, and symbiosis. On the other hand, the combined effect of increased temperature and reduced water availability becomes a powerful filter for existing microbial species (Yuan et al., 2021). The microbial networks in the high-temperature and high-humidity and low-temperature and high-humidity areas also have higher complexity and a higher positive correlation ratio (**Figures 3B, E**). We speculate that the microbial networks respond more to multiple climate factors than to a single one, and cooperative rather than competitive relationships dominate the interactions between microorganisms in the leaf community (Zeng et al., 2025). This indicates a shift in the community strategy, from coping with stress to the collaborative utilization of abundant resources. The humid environment may facilitate the exchange of public goods through diffusion, which is conducive to the formation of collaborative relationships. For instance, ammonia-oxidizing bacteria can provide nitrite to nitrate-oxidizing bacteria, thereby enhancing the nitrogen cycle of plants and supporting the growth of the host under favorable conditions (Yang et al., 2020). Moreover, the VPA analysis showed that the interaction between sunlight and temperature explained up to 50.5% of the fungal community (**Figure 4G**), supporting the view of Zhu et al. on the synergistic effect of climate factors (Zhu et al., 2022).

From an ecological perspective, the microorganisms in the periphery of tobacco leaves adapt to climate change through functional redundancy and dual characteristics of key groups. For instance, high precipitation maintains a high-diversity bacterial community (**Figure 2B**), possibly responding to environmental fluctuations through multifunctional groups; while in low precipitation areas, key groups such as the *Bacteroidetes* maintain metabolic functions (**Figures 2A**, **7**). This strategy is similar to the response patterns of leaf interstitial fungi in alpine grasslands to different precipitation patterns (Li et al., 2023), and responds to different environmental climates by altering community complexity and stability as well as changing the direction of functional metabolic emphasis. Future research can combine with metagenomics to further analyze the molecular regulatory mechanisms of key strains, providing targets for the selection and cultivation of stress-resistant varieties and cultivation. Although this study has achieved insightful results, there are still some limitations that should be acknowledged. Firstly, these functional characteristics were derived by using bioinformatics tools (such as PICRUSt2) to predict the 16S rRNA data. Although this method is very effective, it cannot directly measure gene expression or enzyme activity. Future transcriptomics or proteomics analyses are needed to verify these predicted metabolic functions. Secondly, as mentioned above, a large part of the key functions was classified under the “other” category, which requires a more refined approach to reveal the role of low-abundance taxa (such as metagenomic sequencing). Finally, the inferences regarding plant-microbe interactions are still at the speculative stage; controlled greenhouse experiments are needed to ultimately confirm the causal relationship between the identified microbial species, their functions, and the adaptability of the host plants under climate stress.

## 5 Conclusion

This study employed multi-region sampling and high-throughput sequencing methods to investigate the response of the microbial community on the surface of tobacco leaves to the climate gradient. The research found that there were significant differences in the contents of starch, total sugar, and reducing sugar in the tobacco leaves under different climate gradients, ranging from 26.87% - 32.25%, 14.24% - 16.74%, and 9.96% - 11.26%, respectively. The key climate factors driving the composition of bacterial and fungal communities were completely different. Precipitation was the most dominant factor shaping the structure of the bacterial community (accounting for 41.7% of the variation), followed by the duration of sunlight (accounting for 26.7% of the variation), while the fungal community was mainly regulated by the degree of temperature regulation (accounting for 27.3% of the variation). This independent response indicates that in the leaf-interaction climate adaptation model, the components of bacteria and fungi need to be considered separately. Climate factors have greatly reshaped the coexistence network of microorganisms. In regions with high rainfall, the bacterial community formed a complex interaction network, with the proportion of positive correlations reaching 85.99%, indicating a cooperative strategy under favorable conditions. In regions with less rainfall, the network became simpler (Nodes: 93, Edges: 1124), mainly composed of Sphingomonas (86.50%) and Methylobacterium (10.24%), suggesting that under resource constraints, organisms tend to adapt to stress and reduce ecological complexity. The metabolic potential of the microbial community was reprogrammed with changes in the climate gradient. In regions with less rainfall, the microbial community in the region was rich in genes encoding potential enzymes for starch and sucrose decomposition (such as α-amylase, β-fructofuranosidase), which may enable the stored carbon to be utilized by the host under stress conditions. In regions with high rainfall, the community’s potential for sucrose synthesis (such as through sucrose synthase) was enhanced. This functional division and collaboration is significantly correlated with the changes in the contents of starch and sugar in the leaves, indicating that the leaf-interaction microbial community actively participates in the regulation of the host’s carbon metabolism as a strategy to adapt to climate stress.

## Data Availability

The data presented in the study are deposited in the NCBI repository, accession number PRJNA1393079”; for more information regarding our data policies, refer to our guidelines.
