# Supplementary material for: Regional climate variation structures the phyllosphere microbiome of flue-cured tobacco
Source: Front Plant Sci. 2026 Jan 15;16:1733198. doi: 10.3389/fpls.2025.1733198 (PMC12866982; doi:10.3389/fpls.2025.1733198)
Supplement: Supplementary file 1 [file DataSheet1.docx]

Regional climate variation structures the phyllosphere microbiome of flue-cured tobacco

Cheng Zhang^ab1^, Lei Yang ^c1^ Xiaohua Zhang^a^, Yuhang Zhao^a^, Jiati Tang^b^, Zhijun Cheng^c^, Yi Cao^b^, Shengjiang Wu^b^, Guanhui Li^b^, Long Yang^a*^and Kesu Wei^b*^

^a^College of Plant Protection, Agricultural Big-Data Research Center and Key Laboratory of Agricultural Film Application of Ministry of Agriculture and Rural Affairs, Shandong Agricultural University, Tai’an 271018, China

^b^Guizhou Academy of Tobacco Science; Guizhou Provincial Academician Workstation of Microbiology and Health, Guiyang 550081, China

^c^ China Tobacco Hunan Industrial Co., Ltd, Changsha, 410019 China

^1^Authors to be regarded as Joint First Authors.

^*^Author should be considered a co-corresponding author.

Correspondence: lyang@sdau.edu.cn/weiks8816@163.com

**
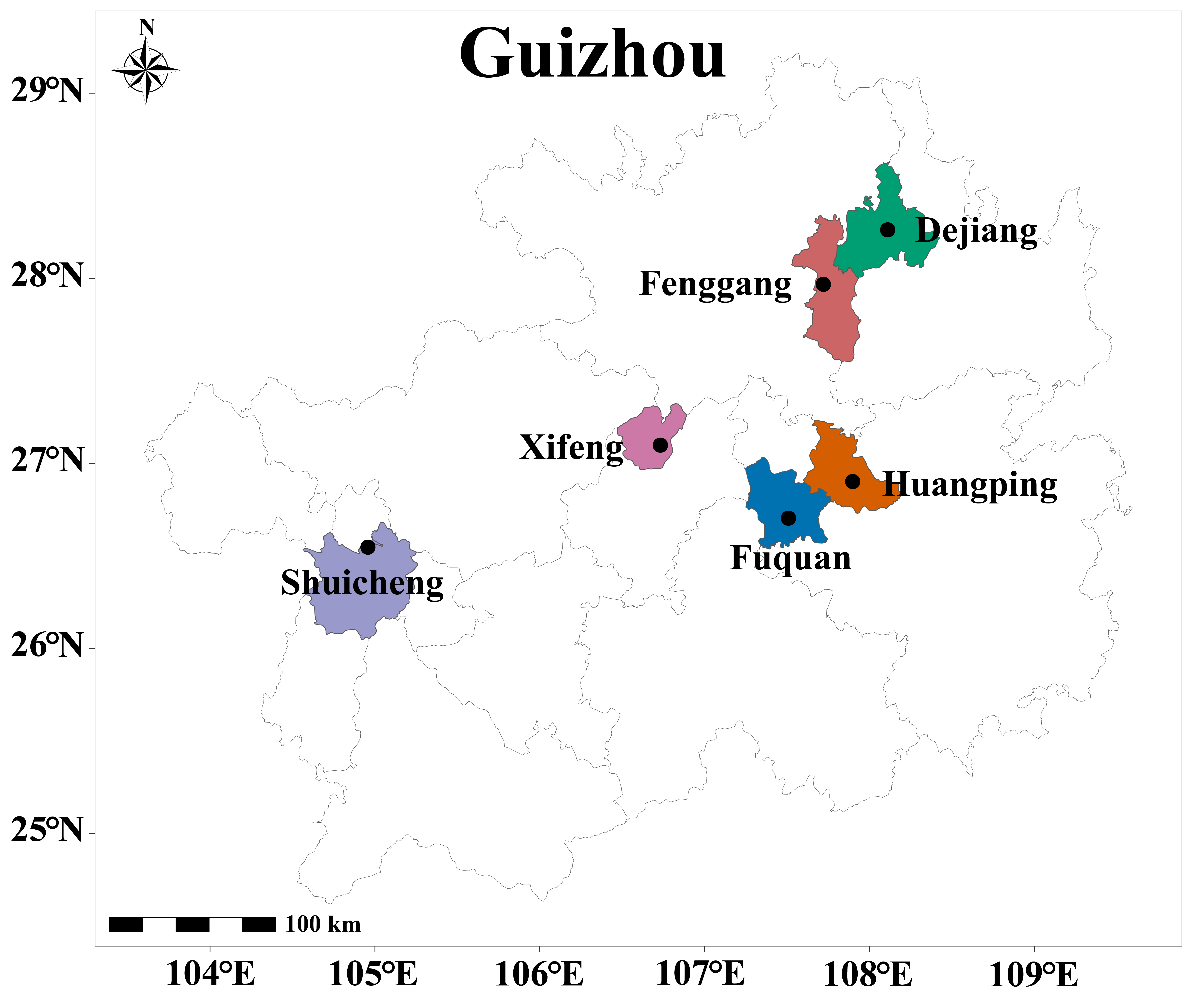
**

Supplementary Figure 1. Six sampling areas, Dejiang (GJ), Fenggang (FG), Xifeng (XF), Fuquan (FQ), Huangping (HP), Shuicheng (SC).

Supplementary Table 1. Climatic Factors in Different Regions in July 2022.

| Environmental factors | DJ | FG | XF | FQ | HP | SC |
| --- | --- | --- | --- | --- | --- | --- |
| Average temperature (AT, ℃) | 27.7 | 27.1 | 25.4 | 26.5 | 26.9 | 21.8 |
| Precipitation  (PR,mm) | 121.8 | 228.5 | 161.9 | 129.3 | 50.6 | 198.6 |
| Sunshine duration  (SD, h) | 278.2 | 246.2 | 261.4 | 267.7 | 231.8 | 190 |
| Effective accumulated temperature (EAT, ℃) | 549.4 | 530.2 | 477.2 | 511.1 | 522.9 | 365.2 |

Supplementary Table 2. The physical and chemical properties of soil at different sampling points.

| Element | DJ | FG | XF | FQ | HP | SC |
| --- | --- | --- | --- | --- | --- | --- |
| Total Nitrogen(g/kg) | 0.15 | 0.13 | 0.14 | 0.15 | 0.14 | 0.17 |
| Total Phosphorus (g/kg) | 0.07 | 0.06 | 0.08 | 0.08 | 0.07 | 0.08 |
| Total Potassium (g/kg) | 2.54 | 2.94 | 2.05 | 2.85 | 2.21 | 2.13 |
| Organic Matte (g/kg) | 24.51 | 27.45 | 23.53 | 26.64 | 22.68 | 27.45 |
| PH | 6.10 | 6.60 | 6.80 | 6.70 | 6.80 | 7.00 |

Supplementary Table 3. Regional Differences in Chemical Components of Fresh Tobacco Leaves.

| Chemical composition | DJ | FG | XF | FQ | HP | SC |
| --- | --- | --- | --- | --- | --- | --- |
| Potassium (K, %) | 2.18±0.31ᵃ | 1.64±0.09ᵇᶜ | 1.84±0.18ᵇ | 1.35±0.05ᵈ | 1.67±0.19ᵇᶜ | 1.77±0.11ᵇᶜ |
| Chlorine (Cl, %) | 0.29±0.04ᵃ | 0.21±0.03ᵇ | 0.25±0.05ᵃᵇ | 0.22±0.04ᵇ | 0.28±0.05ᵃ | 0.23±0.05ᵃᵇ |
| Reducing sugar (RS, %) | 10.88±0.21ᵃᵇ | 10.87±0.32ᵃᵇ | 11.26±0.38ᵃ | 9.96±0.39ᵇ | 10.12±0.66ᵇ | 11.26±0.38ᵃ |
| Starch (ST, %) | 28.35±0.78ᵇ | 31.06±0.67ᵃ | 27.87±0.72ᵇᶜ | 27.69±0.81ᶜ | 26.87±0.73ᶜ | 32.25±0.67ᵃ |
| Alkaloids (TPA, %) | 3.85±0.33ᵃᵇ | 3.90±0.33ᵃ | 3.26±0.25ᵇ | 3.70±0.27ᵃᵇ | 3.52±0.36ᵃᵇ | 3.58±0.19ᵃᵇ |
| Total nitrogen (TN, %) | 2.37±0.31ᵃ | 2.15±0.31ᵃ | 2.45±0.42ᵃ | 2.38±0.29ᵃ | 2.53±0.42ᵃ | 2.43±0.36ᵃ |
| Total sugar (TS, %) | 14.24±0.35ᵇ | 14.60±0.57ᵇ | 15.30±0.73ᵃᵇ | 14.35±0.41ᵇ | 14.67±0.49ᵇ | 16.74±0.38ᵃ |
| Nitrogen-to-alkaloid (TN/TPA) | 0.62±0.14ᵇ | 0.56±0.13ᵇ | 0.75±0.08ᵃ | 0.65±0.13ᵇ | 0.73±0.20ᵃᵇ | 0.68±0.14ᵃᵇ |
| Reducing-to-total sugar (RS/TS) | 1.31±0.06ᵇ | 1.34±0.01ᵇ | 1.36±0.02ᵇ | 1.44±0.02ᵃ | 1.45±0.05ᵃ | 1.49±0.08ᵃ |
| Sugar-to-alkaloid (TS/TPA) | 3.71±0.23ᵇ | 3.76±0.17ᵇ | 4.70±0.13ᵃ | 3.89±0.17ᵇ | 4.21±0.57ᵃᵇ | 4.69±0.35ᵃ |

Note: Based on the results of Duncan's post hoc test, groups with no significant differences are marked with the same letter (p<0.05).

Supplementary Table 4. Regional variation in chemical composition of processed tobacco leaves.

| Chemical composition | DJ | FG | XF | FQ | HP | SC |
| --- | --- | --- | --- | --- | --- | --- |
| Potassium (K, %) | 2.04±0.03ᵇ | 1.49±0.02ᵈ | 1.65±0.07ᶜ | 1.26±0.04ᵉ | 1.56±0.08ᶜᵈ | 1.58±0.03ᶜ |
| Chlorine (Cl, %) | 0.25±0.01ᵇ | 0.17±0.01ᵈ | 0.27±0.01ᵃ | 0.18±0.00ᶜᵈ | 0.22±0.01ᵇᶜ | 0.23±0.01ᵃᵇ |
| Reducing sugar (RS, %) | 23.47±0.49ᵃ | 18.55±0.07ᵈ | 21.23±0.38ᵇ | 14.45±0.23ᵉ | 22.80±0.43ᵃᵇ | 23.22±0.22ᵃ |
| Starch (ST, %) | 4.08±0.16ᵇ | 6.41±0.30ᵃ | 3.71±0.16ᶜ | 4.80±0.31ᵇ | 3.79±0.11ᶜ | 3.67±0.33ᶜ |
| Alkaloids (TPA, %) | 3.41±0.10ᵇ | 3.96±0.01ᵃ | 3.10±0.00ᶜ | 3.52±0.22ᵇ | 3.34±0.23ᵇᶜ | 3.56±0.16ᵇ |
| Total nitrogen (TN, %) | 2.53±0.10ᵃ | 2.02±0.10ᵇ | 2.37±0.10ᵃᵇ | 2.16±0.09ᵇ | 2.29±0.02ᵃᵇ | 2.35±0.19ᵃᵇ |
| Total sugar (TS, %) | 25.27±2.06ᵇ | 25.73±1.67ᵇ | 25.16±0.98ᵇ | 25.06±0.74ᵇ | 26.18±0.38ᵇ | 32.96±1.52ᵃ |
| Nitrogen-to-alkaloid (TN/TPA) | 0.74±0.04ᵇ | 0.51±0.02ᵈ | 0.76±0.03ᵃ | 0.62±0.04ᶜ | 0.69±0.05ᵇᶜ | 0.66±0.08ᵇᶜ |
| Reducing-to-total sugar (RS/TS) | 1.08±0.07ᵈ | 1.39±0.10ᵇ | 1.18±0.03ᶜ | 1.73±0.07ᵃ | 1.15±0.00ᶜ | 1.42±0.07ᵇ |
| Sugar-to-alkaloid (TS/TPA) | 7.42±0.65ᵇᶜ | 6.50±0.42ᵈ | 8.12±0.32ᵃᵇ | 7.14±0.31ᶜ | 7.86±0.45ᵃᵇ | 9.27±0.86ᵃ |

Note: Based on the results of Duncan's post hoc test, groups with no significant differences are marked with the same letter (p<0.05).

Supplementary Table 5. Data parameters of the co-linear network analysis of bacterial and fungal communities between regions.

| Parameter | | DJ | FG | FQ | XF | HP | SC |
| --- | --- | --- | --- | --- | --- | --- | --- |
| Bacterial | Positive corr (%) | 51.63 | 66.16 | 60.09 | 58.89 | 50.53 | 60.99 |
|  | Negative corr (%) | 48.37 | 33.84 | 39.91 | 41.11 | 49.47 | 39.019 |
|  | Node | 216 | 159 | 339 | 381 | 93 | 361 |
|  | Edge | 5497 | 3472 | 13820 | 18861 | 1124 | 13990 |
|  |  |  |  |  |  |  |  |
| Fungal | Positive corr (%) | 51.88 | 52.52 | 54.95 | 58.89 | 53.18 | 85.99 |
|  | Negative corr (%) | 48.12 | 47.48 | 45.05 | 41.12 | 46.82 | 14.01 |
|  | Node | 253 | 220 | 297 | 381 | 279 | 184 |
|  | Edge | 7462 | 7116 | 11433 | 18861 | 10194 | 4461 |

Supplementary Table 6. Permutation test for redundancy analysis of 16S RNA and ITS samples from different regions.

| Explaining variable | Bacterial | |  | Fungal | |
| --- | --- | --- | --- | --- | --- |
|  | r^2^ | p-value |  | r^2^ | p-value |
| Average temperature (AT, ℃) | 0.810 | 0.001 |  | 0.398 | 0.029 |
| Precipitation (PR,mm) | 0.189 | 0.211 |  | 0.637 | 0.002 |
| Sunshine duration  (SD, h) | 0.579 | 0.006 |  | 0.434 | 0.016 |
| Effective accumulated temperature (EAT, ℃) | 0.805 | 0.001 |  | 0.391 | 0.034 |
| Alkaloids (TPA, %) | 0.248 | 0.128 |  | 0.114 | 0.410 |
| Total nitrogen (TN, %) | 0.035 | 0.783 |  | 0.158 | 0.265 |
| Reducing sugar (RS, %) | 0.541 | 0.002 |  | 0.653 | 0.001 |
| Total sugar (TS, %) | 0.344 | 0.040 |  | 0.372 | 0.027 |
| Potassium (K, %) | 0.398 | 0.029 |  | 0.584 | 0.002 |
| Chlorine (Cl, %) | 0.117 | 0.416 |  | 0.348 | 0.041 |
| Starch (ST, %) | 0.337 | 0.042 |  | 0.840 | 0.001 |
